# The effect of food packaging elements on children’s food choices and intake: A systematic review

**DOI:** 10.3389/fnut.2022.998285

**Published:** 2022-12-01

**Authors:** Alazne Arraztio-Cordoba, Rafael A. Araque-Padilla, Maria Jose Montero-Simo, Cristian M. Olarte-Sanchez

**Affiliations:** ^1^Department of Management, Universidad Loyola Andalucia, Cordoba, Spain; ^2^Loyola Behavioral Lab, Department of Psychology, Universidad Loyola Andalucia, Seville, Spain

**Keywords:** packaging, children, food, marketing techniques, nutrition labelling, systematic review, risk assessment

## Abstract

Little can be added about the worldwide concern over the exponential increase in obesity and child overweight problems. Much of the unhealthy eating habits occur at the time of food choice. The enormous influence of marketing strategies in general, and packaging in particular, has been highlighted here. In this respect, public policies that tend to direct choices toward healthier options have been developed. However, the usefulness of such policies will depend on evidence of how different packaging elements can influence children. This systematic review (SR) aims to compile the knowledge available to date on the influence of packaging on food choices and eating behaviours in children. Methodologically, the Preferred Reporting Items for Systematic Reviews (PRISMA) guidelines have been followed to select papers. We also assessed the risk of bias in the studies analysed using the Newcastle-Ottawa Quality Assessment Scale (NOS). The initial search strategy found 2,627 articles, although only 20 of them met the eligibility criteria. Data from the studies were extracted, categorised, and analysed. The results indicate that most of the packaging elements have some effect on children’s food choices or food intake. The use of Cartoon is the element with the most consistent evidence of influence. Despite the number of studies and public initiatives developed to promote this informative and persuasive element, less consistency has been found regarding the effect of Nutrition Labelling. Therefore, the results found should be considered by both governments and organisations when promoting public policies that work for the wellbeing of children.

## Introduction

Marketing is present daily in many facets of our lives. Among its various objectives, it seeks, through different management techniques, to capture the desired target’s attention and interest to improve sales opportunities (1). This is true for food products aimed specifically at children (2). The food industry is one of the most active marketing tools, as it operates on a saturated and competitive market (3). For this reason, marketing is essential, as it is the primary differentiating element (4, 5). Traditional mass media campaigns or the persuasive use of product packaging attract children’s attention (6). In particular, in continuous evolution and growth, the packaging strategy has become an element that reinforces the commercial appeal of food products aimed at children (4, 7). In these cases, its use has been socially questioned when it comes to unhealthy products [according to the recommendations of the World Health Organization (WHO)], a category exceptionally dynamic in the use of commercial persuasion (8, 9). This concern has to be understood in a global context of an alarming exponential increase in childhood obesity [38.9 million overweight children in 2020 (10, 11)], with serious doubts about the possible impact on the long-term health and full development of children.

Packaging has been studied in the literature to shed light on its possible impact on purchasing decisions. Thus, in the case of children, we find systematic reviews (SRs) where packaging is studied together with other marketing tools, such as advertising or product placement, according to its effect on eating behaviour (12–15). Previous, more specific reviews on the packaging either do not address its effect on children (16) or only analyse the effect of more visual marketing appeals (8). The review by Hallez et al. (17) analyses the effect of each packaging element on food choice and food intake, comparing children and adults. However, this is a separate analysis of each element without analysing what happens when elements of a different nature are combined in the same packaging.

This is precisely the main objective of this SR, to highlight the current knowledge on how the different packaging elements interact and how the effects of some elements can be annulled or enhanced in the presence of others. Especially if we find ourselves with elements of a very different nature, as in the case of the combination of persuasive or attractive elements versus more informative or dissuasive elements (13). This has led us to differentiate the elements to be studied between Nutrition Labelling and Marketing Techniques.

Nutrition Labelling is responsible for informing consumers about the nutritional properties of food. They were created to help consumers to make more healthy purchasing and consumption decisions, to avoid misleading labelling, to protect their health, and to ensure fair practices in the food trade (18). Addressing its informative appeal, we considered Front-of-pack Nutrition Labelling (FOPL) and the Nutrition FACTS Label (FACTS) as part of Nutrition Labelling. The FOPL is a type of graphic labelling intended to help consumers improve their understanding of nutritional information on food products [e.g., Traffic Light System (as Nutri-Score), Nutritional Warnings, Guideline Daily Amount, Reference Intake, or Health Star Rating System]. Furthermore, FACTS is information provided to the consumer about the nutritional profile of foods. It is generally quantitative and is intended to convey an understanding of the number of nutrients in a product (19).

Marketing Techniques are considered persuasive techniques used by the food industry to attract children’s attention, improve product recognition, and create a positive attitude toward the brand. Attending the scope of this study, we have considered as Marketing Techniques visual elements such as cartoons or characters, tie-ins with movies or TV shows, games and advergames, giveaways, child-appealing imagery or graphics, product shape, colour, products packaged explicitly for children, and serving suggestion image (16). Moreover, all types of Claims are considered Marketing Techniques, because, although they are not visual elements, they generate attractiveness to the product. Even if they are regulated by The Codex Alimentarius to protect consumers from false and misleading messages (20), the food industry makes voluntary use of them as an appealing element.

Taking into account all these different but complementary intentions of the packaging elements, the comparative and contrastive study of Marketing Techniques and Nutrition Labelling is therefore indispensable. Only by knowing how the different packaging elements interact with each other, and the effects of one on the other, we will be able to fully understand the real impact of products aimed at children. An impact that could affect the most vulnerable sector of the population misleading them to do unhealthy decisions.

To do so, is necessary to examine all the elements of packaging, covered in the previous literature, with the aim of finding out their effect on food choice and food intake. For this reason, this SR has as preliminary objective:

(I) To find out the effect of each packaging element separately as discussed in the literature on children’s food choices and/or food intake, whether they are Marketing Techniques or Nutrition Labelling elements.

Moreover, to understand the interactions among elements, this SR pursues the following main research objective:

(II) To find out the effect of different packaging elements in the presence of other types of elements on children’s food choices and/or food intake; and thus to see if the effect in isolation is modified in the presence of other packaging elements, especially when it comes to the interaction of elements that could generate some cognitive dissonance.

This will be done by reviewing systematically previous experimental articles, which allow the almost total control of the study variables according to the objective pursued, the identification of the cause-effect relationship, and the replicability and testability of the studies. In addition, these experiments should yield quantitative results to ensure that confirmatory effects can be obtained (21) and will be considered according to the methodological quality of their experimental procedures with the ultimate aim of yielding more reliable conclusions, avoiding heterogeneity, and guaranteeing the consistency of the results. These will be analysed with particular attention to possible risks of bias, something that has not been fully taken into account in other SRs on packaging.

The results will provide relevant information for policymakers, programme managers, and health professionals to design public policies aimed at a healthier diet for children.

## Methods

This review followed the Preferred Reporting Items for Systematic Reviews (PRISMA) guidelines (22, 23) (see Supplementary Tables 2, 3 for PRISMA 2020 Checklist)”.

### Eligibility criteria

The PICOS (Population, Intervention, Comparator, Outcome, Study design) eligibility criteria were as follows (24):

#### Population

Participants must be children and adolescents <18 years old. Adults over 18 years of age are therefore excluded.

#### Type of study

The articles included those focussed on the packaging of food aimed at children, analysing its different elements as a whole or separately: Marketing Techniques on Packaging (e.g., Colour, Shape, Size, In-product Promotions, Celebrities, Claims as Nutrition Claims or other Claims) and Nutrition Labelling (e.g., FOPL and FACTS). Therefore, any study that analyses other marketing tools such as TV commercials or videos on digital platforms is excluded.

#### Comparators

The comparators took into account as inclusion criteria those focussed on evaluating the Marketing Techniques present on the Packaging of food products aimed at children: Outcome with neutral packaging, outcome toward a product with a given packaging element versus another, and outcome with or without intervention.

#### Outcomes

Two categories of outcomes were considered: (I) Food choice and (II) Food intake.

#### Study design

The study designs are experimental studies with or without intervention (randomised and non-randomised controlled, with a control group) that offer quantifiable results. For those studies with intervention, there is no fixed time of the action or follow-up period for the experimental and control groups. Those experiments that work with qualitative methodologies (e.g., Focus groups, interviews) are excluded from our study. The studies may have been conducted in the laboratory, field, or hybrids. Moreover, studies conducted on online platforms have also been taken into account.

#### Others

No restriction was placed on the year of publication of the papers chosen. Nor are the studies restricted by geographic area or country income level. All papers included must have been written in English. Any publication other than a journal article (e.g., conference abstracts, web articles, editorials) is excluded.

### Information sources and search strategy

The initial literature search was conducted in six databases in November 2021. The databases were as follows: MEDLine, Academic Search Ultimate, Business Source Ultimate, PsycINFO, Cochrane Library, Web of Science.

The string of terms used for the paper search was as follows: (”food package*” OR “food label*”) OR (”nutrition label*” OR “nutrition fact*”) AND (child* OR “youth” OR “young” OR adolescent* OR school* OR preschool* OR teen*).

For each database, the search was configured to be performed on the abstracts of the articles (AB). Thus, this search was systematised to obtain a set of articles from which duplicates had to be eliminated. After this, two fundamental steps were followed to locate articles included in SR: Reading the title and abstract of the papers and then reading them in full. The first phase was carried out by AA-C, RG-C, and MS-T, and the second phase by AA-C and CO-S. The PICO structure considered the inclusion and exclusion criteria in both phases. Reading the title and abstract took approximately 1 month and was carried out independently and blindly by the reviewers. In comparing the results obtained, an agreement rate of 95% was reached. A disagreement review was required until a 100% agreement rate was reached. After this step, the potential full-text articles were searched for and read by AA-C and CO-S, again in a blinded and independent manner. This process took a month and a half. Pooling the results resulted in substantial agreement (kappa = 0.62, 95% CI 0.40–0.85). Disagreements were discussed to determine the total number of articles included in the SR.

### Quality assessment

The tool used to evaluate the articles included in the SR was the Newcastle-Ottawa Quality Assessment Scale (NOS) (25); specifically, the tool created to evaluate Case-Control Studies. NOS uses three main categories to assess the studies: Selection, Comparability, and Exposure. Each category has a series of items accompanied by stars. The sum of the set of stars determines whether a study has a low risk of bias (>7 stars) or, conversely, a high risk of bias (<7 stars). To complete the results of the NOS tool, the free version of the Revman 5 software (Review Manager) developed by The Cochrane Collaboration was used to extract the Risk of bias graph, which helps to interpret the results quickly and visually.

### Charting the data

After analysing the Risk of the articles, a standardised extraction of the relevant data from the included studies was performed. This data extraction was also carried out with the participation of the two reviewers (AA-C and CO-S), without conflicts. The extracted data were divided into two tables, the first with the key information of the studies and the second with the results. The extracted information is as follows:

- General information and identifiers of the studies: (authors and year of publication).
- Study population data: (sample size, target, median age, and region).
- Study tools: (type of experimental design, nutritional value of the food, assessment tool, and existence of intervention).
- Comparators of the study: (detail of the experimental groups, control groups, stimulus materials, Packaging Marketing Technique).
- Outcomes of the study: (food choice and food intake and their effect).

### Collating, summarising, and reporting the results

The main characteristics of the studies analysed have been synthesised in a table that summarises information about the sample sizes of the experiments, the regions of performance, the quality of the studies, the nutritional information of the foods, and the prevalence of these in the experiments, the type of experimental design, whether or not there is an intervention in the studies, the outcomes reported, and the packaging element analysed. This table provides a descriptive and global perspective of the results of this SR.

## Results

### General description of the article selection

A total of 2,627 articles were initially extracted from the databases. After eliminating duplicates, 1,289 articles were eligible for reading by title and abstract. Of these, 64 articles were considered for a full reading. In the complete reading, 21 articles were found that did not meet the methodological requirements (lack of control group, qualitative experiments, or non-experimental procedures), and 7 articles were eliminated because their objective did not fit the one intended in this SR (they did not study packaging), 9 articles were eliminated because the population did not fit the PICO structure of our study (adults) and, finally, 10 articles were eliminated because the outcome did not correspond to food choice or food intake. A backward search of reference lists led us to include 3 more articles.

**FIGURE 1:**
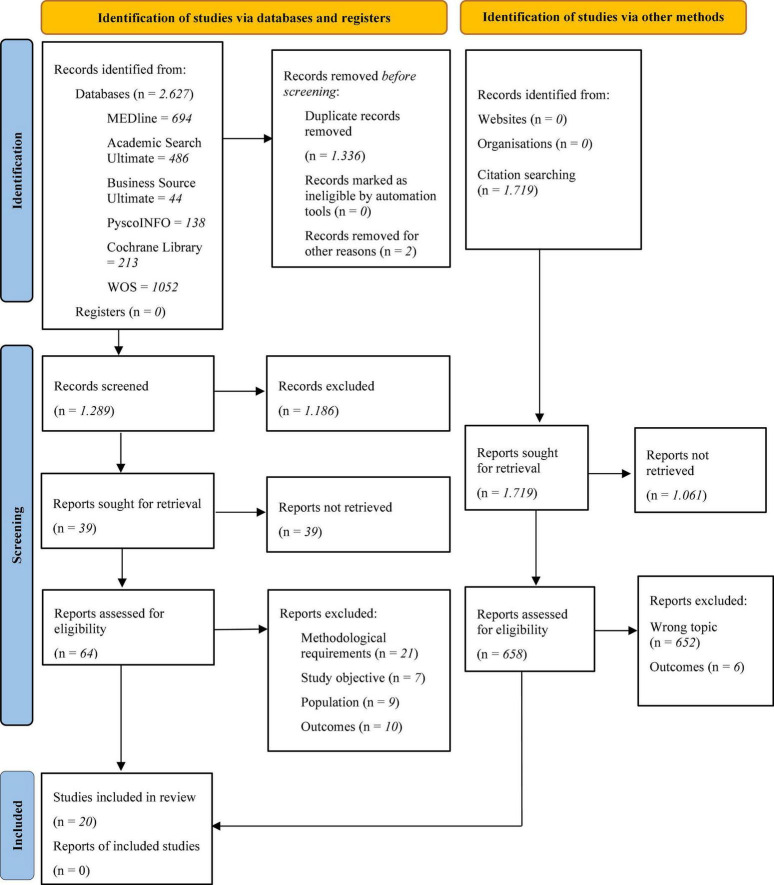
Preferred reporting items for systematic reviews (PRISMA) flow diagram of the included and excluded studies throughout the systematic review protocol (23).

Finally, 20 studies met all the SR inclusion criteria. Figure 1 shows the PRISMA Flow diagram (23), which schematises this article’s process of obtaining the papers.

### Study characteristics

**FIGURE 2:**
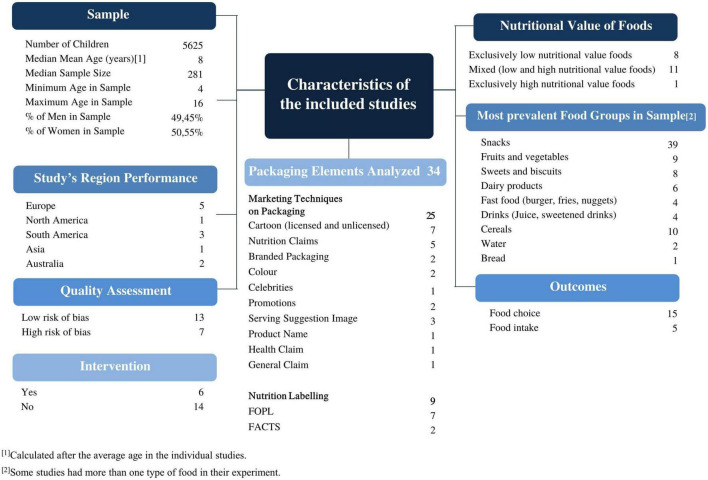
Key characteristics of the included studies.

The key characteristics of the included studies are summarised in Figure 2. The most relevant results are presented in the following paragraphs (see Supplementary Table 1 for further characteristics of included articles).

#### Country, year, and experimental design of the studies

There are differences in the date of the studies (the range is from 2010 to 2020). The years with the most publications were 2014 (26–28) and 2019 (29–32), with an increasing trend in recent years. The regions where the studies were conducted are detailed below: North America: USA (29, 33–39), Canada (40); Europe: UK (41, 42), Belgium (32, 43), Iceland (44); South America: Brazil (31), Guatemala (27), Uruguay (45); Australia (26, 28), and Asia: Indonesia (30). In terms of experimental design, most of the articles present a Between-Subjects design (55% of the studies) (26, 28, 29, 31, 33–36, 39, 40, 42); the rest present a Within-Subjects design (35%) (27, 32, 37, 38, 43–45); a single article presents a Mixed-measures design (41) and another a quasi-experimental design (30). Six studies are experiments with intervention. These interventions were educational, and their main objective was to teach children about nutrition and healthy eating habits. Three of them are focussed on instructing children to read and interpret Nutrition Labelling properly and to get them to make an appropriate and autonomous choice of the foods they will acquire and consume (29, 30, 36). One of them, using short nutrition lessons and different marketing techniques such as colour, cartoons, and promotions, tries to encourage children to consume fruits and vegetables (35). Another one focussed on how counter-advertising teaches children about healthy food choices (26). The last one teaches children key aspects of nutrition and physical activity (39).

#### Nutritional quality of food products

The nutritional quality of the foods present in the experimental procedures of the studies has been categorised into low, mixed and high nutritional value, according to WHO recommendations (46). Thus, low nutritional value products are highly processed, with ingredients that contribute to the intake of saturated fats, sodium, and sugars (e.g., sweets, sweet drinks, snacks, or cereals). A total of 40% of the studies analyse products exclusively with a low nutritional value. Furthermore, 55% of the articles studied foods with both low (unhealthy) and high nutritional value (healthy). These articles are referred to as mixed nutritional value. Only one study uses foods with high nutritional value, which are products that are recommended by the WHO to follow a healthy diet (water, bread, fruit juice, yoghurt, and carrots) (44).

#### Risk of bias

**FIGURE 3:**
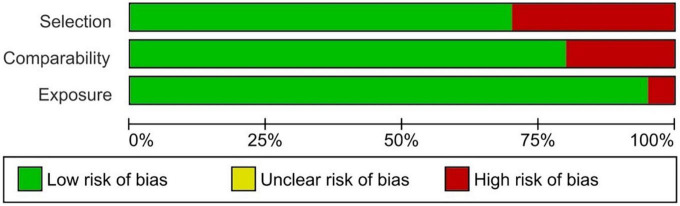
Risk of bias graph.

A total of 65% of the studies included in this SR show a low risk of bias in terms of Selection, Comparability, and Exposure (see Table 1 and Figure 3). A high risk of bias was found in six studies (31, 35, 37–40, 44) which will be considered when analysing them because of the possible inconsistency of their effects and the heterogeneity of their results.

**TABLE 1:** Quality appraisal studies.

References	Selection	Comparability	Exposure	Conclusion
Aerts and Smits (32)	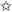 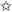 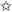 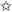	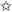	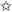 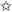	Low risk of bias
Arrúa et al. (45)	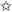 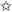 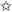 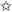	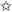	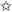 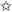	Low risk of bias
Becker et al. (29)	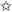 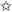 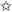	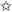 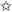	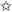 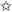	Low risk of bias
Dixon et al. (28)	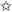 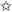 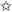	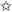 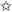	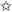 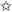 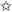	Low risk of bias
Dixon et al. (26)	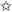 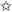 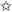	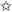 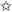	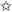 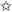	Low risk of bias
Elliot et al. (40)	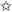 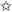	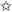 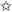	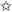 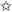	High risk of bias
Gunnarsdottir and Thorsdottir (44)	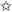	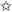 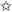	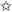 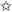	High risk of bias
Heard et al. (34)	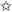 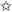 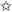	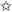 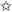	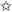 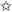	Low risk of bias
Katz et al. (36)	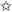 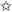 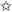	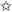 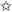	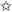 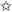 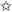	Low risk of bias
Keller et al. (35)	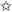	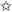 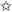	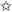 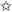	High risk of bias
Lapierre et al. (33)	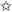 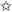 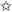	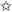 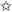	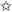 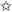	Low risk of bias
Letona et al. (27)	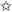 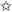 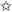 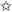	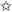 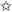	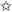 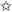	Low risk of bias
Lima et al. (31)	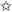 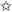 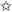	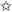	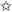 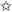	High risk of bias
McGale et al. (41)	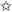 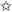 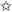	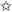 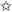	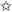 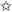	Low risk of bias
McGale et al. (42)	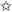 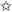 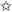 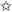	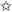 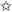	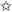 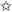	Low risk of bias
Miller et al. (39)	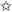 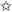	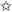	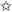 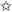	High risk of bias
Neyens et al. (43)	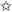 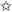 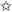	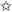 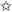	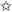 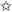	Low risk of bias
Retno (30)	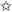 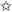 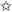 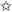	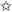 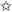	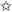 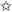	Low risk of bias
Roberto et al. (37)	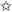 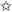	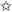 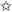	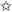 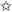	High risk of bias
Soldavini et al. (38)	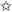 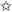	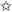 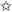	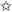	High risk of bias

### Main findings

In the following section, the results of the selected studies will be shown. We will develop the results about the different packaging elements, considering the quality of the studies, the nutritional value of the foods they accompany, and their effectiveness on food choice and food intake in children (see Table 2 for the key results of included studies).

**TABLE 2:** Key results of included studies.

References	Study region	Total sampling size	Packaging elements	Nutritional value of food	Outcome measure	Effect obtained
Aerts and Smits (32)	Europe (Belgium)	71	Serving suggestion image	Mixed	Food intake	Children intake more food when the serving suggestion image is more extensive. A greater effect is found in healthier foods.
Arrúa et al. (45)	South America (Uruguay)	221	Cartoon, nutrition claims, FOPL guideline daily amount, FOPL traffic light	Mixed	Food choice (preference)	Children choose food with cartoons regardless of income.
Becker et al. (29)	North America (United States)	80	FOPL traffic light	Low	Food choice (preference)	FOPL with traffic light system helps children choose healthy products (regardless of their age). Training improves these results.
Dixon et al. (28)	Australia	1,351	Nutrition claims	Low	Food choice	Counter-advertising (type of training) moderates the effect of nutrition claims and helps children choose healthier foods.
Dixon et al. (26)	Australia	1,302	Nutrition claims, celebrities, premium offers	Low	Food choice (preference)	Children choose products with nutrition claims and celebrities (sports celebrity endorsements influenced boys’ but not girls). Premium offers have no impact on them (boys and girls).
Elliot et al. (40)	North America (Canada)	65	Colour, branded packaging	Mixed	Food choice (identification)	Children choose the taste of foods with coloured packaging rather than branded packaging.
Gunnarsdottirand Thorsdottir (44)	Europe (Iceland)	66	Cartoon	High	Food choice (preference)	Children choose food with cartoons rather than regular packaging.
Heard et al. (34)	North America (United States)	61	In product–promotion	Mixed	Food choice (identification)	Children choose the products with the best flavour before those that contain promotions.
Katz et al. (36)	North America (United States)	1,180	FOPL, FACTS (undetermined type)	Mixed	Food intake	FOPL and FACTS do not help to reduce children’s BMI. Training helps to improve their understanding of both types of nutrition Labelling.
Keller et al. (35)	North America (United States)	103	Cartoon, colour, branded packaging	Mixed	Food intake	Study 1 = Branded packaging does not increase food intake (OW and non-OW children studied together). OW children consumed more food with branded packaging compared with non-OW.
						Study 2 = Children eat more food with Cartoons regardless of their weight status and the healthiness of the product.
						Study 3 = Children eat more food with cartoons and colours on their packaging than others without those marketing techniques on packaging.
Lapierre et al. (33)	North America (United States)	80	Cartoon, product name	Low	Food choice (taste)	Cartoon and product name influence children’s food choices. Children choose cartoons more than products with product names.
Letona et al. (27)	South America (Guatemala)	121	Cartoon	Mixed	Food choice (preference)	Children choose food with a cartoon rather than clear packaging.
Lima et al. (31)	South America (Brasil)	400	FOPL traffic light, FOPL nutritional warnings	Low	Food choice	FOPL helps children make healthful choices but does not modify their choices when they taste the products.
McGale et al. (41)	Europe (United Kingdom)	209	Cartoon	Low	Food choice (preference)	Children choose food with a cartoon (congruent or incongruent).
McGale et al. (42)	Europe (United Kingdom)	41	Serving suggestion Image	Low	Food intake	Children intake more cereals when the serving suggestion image is more extensive.
Miller et al. (39)	North America (United States)	124	Nutrition claim, health claim, general claim	Mixed	Food choice	Children choose unhealthier products when on-package claims are present. Intervention reduces its impact.
Neyens et al. (43)	Europe (Belgium)	22	Serving suggestion image	Mixed	Food intake	Children intake more cereals and milk when the serving suggestion image is more extensive, A greater effect is found in foods with low sugar content.
Retno (30)	Asia (Indonesia)	41	FOPL traffic light, FACTS	Mixed	Food choice (preference)	FOPL with traffic light system helps choose healthier products better than FACTS.
Roberto et al. (37)	North America (EEUU)	40	Cartoon	Mixed	Food choice	Children better choose foods that have cartoon on the packaging. The effects are weaker on healthy foods than on less healthy foods.
Soldavini et al. (38)	North America (EEUU)	47	Nutrition claims	Low	Food choice (preference)	Children choose the product with nutrition claims as being healthier and tasting better.

#### Packaging elements analysed

A total of 73,53% of the packaging elements analysed in this study correspond to Marketing Techniques on Packaging, compared to 26,47% of Nutrition Labelling. The Marketing Techniques on Packaging found in the studies, according to their prevalence, are: Cartoon (27, 33, 35, 37, 41, 44, 45), Nutrition Claims (26, 28, 38, 39, 45), Serving Suggestion Image (32, 42, 43), Branded Packaging (35, 40), Colour (35, 40), Celebrities (28), In-product Promotions (34), Premium Offers (28), Product Name (33), Health Claim (39), and General Claim (39). For Nutrition Labelling, FOPL —especially Traffic Light System (29–31, 45), Nutritional Warnings (31), Guideline Daily Amount (45), and one of them, with an undetermined type (36)— followed by FACTS (30, 36) stands out. In the experimental design of the articles, these elements are studied in some cases in isolation and others in comparison with other elements. In the case of Cartoons, they are analysed in isolation in 57.14% of the cases (*n* = 4), compared to 42.86% in combination. Promotions and FACTS appear in isolation in half of the articles in which they are analysed (*n* = 1). In the case of FOPL, they are analysed in isolation in 40% of the articles reviewed (*n* = 5), and Nutrition Claims in 40% of the cases (*n* = 2). Elements such as Branded Packaging, Colour, Celebrities, and Product Name are analysed in combination with other packaging elements in all the articles in which they are included. The Serving Suggestion Image is the only element not studied in combination with other packaging elements.

Considering all the packaging elements together we observe that the most prevalent is the Cartoon, licenced or unlicensed, being present in 35% of the articles reviewed (57.14% with a low risk of bias). The following most prevalent elements are FOPL, present in 25% of the articles (80% with a low risk of bias), and Nutrition Claims, present in 25% of the articles too (75% with a low risk of bias). It is worth mentioning that FOPL has been studied with different presentation formats, for example, between Traffic Light Systems with other formats such as Guideline Daily Amount and Nutritional Warnings (*n* = 7 but in 5 studies).

Cartoon has been studied in 28.57% of cases in foods with low nutritional value, compared to 40% in the case of FOPL and 60% in the case of Nutrition Claims.

#### Elements effectiveness asset across studies

We will begin by analysing the evidence found regarding the effect of Marketing Techniques on Packaging and then focus on Nutrition Labelling.

Within Marketing Techniques on Packaging, specifically in the case of Cartoons, we can state that the evidence of their effects is consistent, as all studies find a positive influence of Cartoons on both food choice and food intake (27, 33, 35, 37, 41, 44, 45). However, most of the articles have focussed on food choice (*n* = 6) (27, 33, 37, 41, 44, 45) and 67% of them have a low risk of bias (27, 33, 41, 45), supporting the consistency of the results. On the other hand, the results for food intake should be taken with caution, as they have a high risk of bias, and no studies are considered in this SR to contrast these findings.

Only one comparative analysis of the Cartoon with other packaging elements has been found. This is the case of Lapierre et al. (33), which compares it with the Product Name. Their results conclude that Cartoons have a more significant influence on children’s food choices. There are also no conclusive effects on the greater or lesser power of Cartoons according to the healthiness of the food. Although Roberto et al. (37), found evidence of a greater effect of Cartoons on unhealthy foods versus healthy foods, the study suffers from a high risk of bias.

For the Nutrition Claims, there is evidence that it favours children’s food choices, but not as consistently as in the case of Cartoons. Although more studies have been found that demonstrate its effectiveness (*n* = 4) (26, 28, 38, 39), most of them with a low risk of bias. There is also a study with high methodological quality that fails to demonstrate these effects (45). On the other hand, no evidence has been found on the influence of Nutrition Claims on food intake.

Regarding the Serving Suggestion Image, all the studies demonstrate its effectiveness in children’s food intake (32, 42, 43). As those studies have a low risk of bias (*n* = 3), we can confirm the consistency of these results. There are no studies that demonstrate its effectiveness in food choice.

As far as Branded Packaging is concerned, no solid results have been found on its effects on food choice or food intake in children. Of the two studies with a high risk of bias, one shows its effectiveness on food choice (40), and the other fails to prove its effect on food intake (35). Given the scarcity of scientific evidence with high methodological quality to support these results, we cannot conclude the influence of this element. Similar conclusions were reached with the results for Colour. This packaging element seems to affect food choice and food intake in children. Still, both studies demonstrating this have a high risk of bias, which is evidence of the heterogeneity of these results (35, 40). In a comparative study between Branded Packaging and Colour and their impact on food choice, Elliott et al. (40) found greater Colour effectiveness than Branded Packaging. However, this study also presents a high risk of bias, so its results should be taken with caution due to their possible inconsistency.

On the other hand, there is also an effect of Celebrities, specifically Sports Celebrities, on food choice in boys, although the same effect could not be demonstrated in girls (28). No studies have been found that prove their effectiveness on food intake.

In the case of Promotions, and with the caution of the low number of studies found, we can determine, with certain reliability thanks to the low risk of bias of the studies (*n* = 2), that they have no effect on food choice in children (28, 34).

Regarding Product name, there is evidence of high methodological quality that supports its effect on food choice, but in a limited way with only one study in this respect (33).

With regard to Nutrition Labelling, some inconsistency has been found in the results of the articles using FOPL. While two articles with high methodological quality found a positive impact on food choice in children (29, 30), another two, one of them with high methodological quality (45), and another with a high risk of bias (31) did not find a significant impact on food choice. However, it seems to help, if the children have more excellent knowledge about the product’s healthiness. The only article, which also presents a low risk of bias, indicates that the FOPL does not affect children’s food intake (36). Consequently, with these results it is difficult to establish a significant influence of this packaging element, therefore more research is needed on this element. This is of great relevance if we consider that its implementation in packaging is recommended by the European Commission (EC) (47). Regarding FACTS, there is evidence of an effect on children’s food choices in only one article with a low risk of bias (30). The same does not happen with its impact on food intake since the study that tests them does not yield conclusive results (36). The article by Retno (30) compares the effect of FOPL and FACTS, where the effect of the former type of labelling is greater than the latter type. However, the children in this study are adolescents (mean = 16). More experiments should be carried out to corroborate this with younger children.

Some studies show an analysis of possible important variables; sociodemographic (Income, Gender, Age, and BMI), intervention (counter-advertising or training) and healthiness of a product (sugar) which contribute to moderate the results found. Regarding sociodemographic variables, gender has been found to have a moderating effect in studies on Sports Celebrities. In the study by Dixon et al. (28) a higher probability of choice is evidenced in boys versus girls. The Body Mass Index (BMI) also produces a moderating effect on the influence of Branded Packaging. This effect increases food intake as the child’s BMI increases (35). However, this last finding should be reviewed with caution due to the high risk of bias in the study. Conversely, income does not moderate the effect of the packaging elements analysed (Nutrition Claims, FOPL, or Cartoons) (45). Neither does age in the case of FOPL (29). Furthermore, product healthiness has been found to have a moderating effect on studies observing the effects on Serving Suggestion Image. In 2/3 of the studies (with low risk of bias), the less healthy the product was, the more effect on food intake Serving Suggestion Image has.

We can point out the intervening variables that both moderate the effect of some packaging elements. In the case of counter-advertising, these mediate the impact of the Nutrition Claims; the more significant the presence of counter-advertising, the lower the impact of the Nutrition Claims (26). In this case, the counter-advertising was intended to help the child not to choose unhealthy foods, even though they were packaged with Nutrition Claims. About the training, we have been able to determine, thanks to several studies, that it produces a moderating effect on FOPL and that the more training (through health lessons, for example), the greater the children’s understanding of this element of the packaging (29, 30, 35, 36).

## Discussion

The present SR established a series of research objectives that aimed to synthesise the existing evidence on (I) The effect of each packaging element separately on children’s food choices and/or food intake, whether they are Marketing Techniques or Nutrition Labelling elements and (II) The comparative effect of packaging elements (Marketing Techniques and Nutrition Labelling) on children’s food choices or food intake. The findings obtained will be discussed below.

Firstly, it should be noted that there is little scientific evidence on the effect of different packaging elements on food choice and, especially, food intake in children. The relatively low number of studies becomes even more pronounced when we focus on particular packaging elements, as not all of them have received the same attention. Furthermore, this evidence is sometimes at high risk of bias, leading to inconsistencies in the results. However, it should be noted that in our study, 65% of the studies have a low risk of bias, which allows us, with the necessary caution, to draw some conclusions with a certain degree of robustness.

We can conclude with more consistency, due to their greater prevalence in the studies, the effect of three packaging elements: Cartoon, FOPL, and Nutrition Claims, albeit to varying degrees. As stated above in the results, the presence of Cartoon has been the most studied and on which there are more solid conclusions. In this sense, all the studies reviewed highlight a direct influence on children’s food choices and food intake. In the latter case, there is only one piece of evidence with a high risk of bias. More doubtful is the influence that FOPL and Nutrition Claims may have, with contrary evidence in both cases; although, in the case of FOPL, there are more studies where no effect on food choice and food intake is found. In analysing the evidence for FOPL and Nutrition Claims, it is necessary to consider a moderating element of their results: the knowledge transmitted to the child about the nutritional properties of the product.

Considering the results, we found certain similarities with the SR by Smith et al. (15), Hallez et.al. (17), and Elliot et al. (8), but also certain discrepancies. Our study agrees that the Cartoon is the most prevalent and clearly effective element. Elliot et al. (8), do not show results on the effectiveness of elements such as FOPL, FACTS, or Nutrition Claims, but they concluded that children pay more attention to them than to Cartoon. Our review cannot shed light on this since we do not have any study comparing the influence of these elements. Hallez et al. (17), with little evidences, seem to intuit the scarcity of the effect of FOPL on food choice and food intake in children. In our study, we found more evidence that seems to support these findings.

The low prevalence of the other packaging elements analysed makes us cautious with the conclusions reached in different studies. Elements such as Colour, the use of Celebrities, Branded Packaging, Serving Suggestion Image, and Product Name seem to have a positive influence on food choice. However, it is necessary to have a greater number of evidence and to improve, in some cases, the methodological quality of the studies. Elliot et al. (8), also mention their effectiveness, although with little methodological precision in the case of some elements such as Colour and Branded Packaging. The use of Promotions and Serving Suggestion Images deserve a separate mention. Considering the scarce evidence found, all the articles that analyse Promotions are conclusive about their lack of impact on children’s food choices. And, in the case of Serving Suggestion Image, and concurring with the findings of Hallez et al (17), we can conclude that Serving Suggestion Image has an effect on children’s food choices, especially in healthier foods.

Another problem related to the second objective of our work is the lack of comparative studies between the different packaging elements. The evidence found so far does not allow us to conclude the overall effect of other packaging elements. That is, in the presence of different elements, which are the ones that define the child’s choices or which combination of them is the most influential.

The deficiency of comparative studies was also pointed out by Elliot et al. (8), highlighting the lack of certainty regarding which is the most relevant packaging element in the presence of others since it seems not to have been able to analyse comparisons of effectiveness between various elements, nor the full spectrum of existing ones. Finally, the authors reflect on the incomplete image of the power of packaging in current literature, fundamentally due to the study of the elements in isolation.

The results obtained allow us to support some of the policies developed by the EU on the recommendations for the use of Nutrition Labelling and Nutrition Claims [Regulation (EU) No. 1169/2011 of the European Parliament and of the Council (25 October 2011) on the provision of food information to consumers, amending Regulations (EC) No 1924/2006 and (EC) No 1925/2006 of the European Parliament and of the Council, and repealing Commission Directive 87/250/EEC, Council Directive 90/496/EEC, Commission Directive 1999/10/EC, Directive 2000/13/EC of the European Parliament and of the Council, Commission Directives 2002/67/EC and 2008/5/EC and Commission Regulation (EC) No 608/2004 (48), or Commission Regulation (EU) No 1047/2012 of 8 November 2012 amending Regulation (EC) No 1924/2006 with regard to the list of Nutrition Claims (49)]. The experimental studies analysed do not allow us to conclude on the effectiveness of this type of element on packaging to promote healthier eating in children. On the one hand, experimental studies on the influence of such elements show contradictory results. There is no consistent evidence of their impact. On the other hand, we do not know how Nutrition Labelling influences the presence of other Marketing Techniques on Packaging, which may introduce dissonant information. There is evidence about their attractiveness for children’s choices. Finally, it seems that the effectiveness of Nutrition Labelling and Nutrition Claims is conditioned by prior nutritional knowledge or induced by the opinions of other influential actors for children. The effect of Nutrition Labelling may likely be shallow in less vulnerable families with lower educational levels or less exposed to social media.

Finally, with all the limitations and caveats noted above, we have some evidence, on how the use of different Marketing Techniques on Packaging improves the attractiveness of products. So far, as indicated above, most of these techniques have been used for products of low nutritional value. It might therefore be suggested that these techniques could also be used to encourage the choice of other healthier products through a more attractive packaging design.

The study presented here has the strength of having assessed the risk of bias in the studies analysed. The risk assessment has been considered to draw conclusions. However, the difficulty in finding statistical data (such as effect sizes) in the studies reviewed, as well as their heterogeneity in terms of the results provided, has not allowed us to propose a meta-analysis of the study, which would have allowed a statistical comparison of the results obtained in the different studies.

Additionally, the present review has the strength of having focussed on studying the comparative effects between Marketing Techniques on Packaging and Nutrition Labelling on children’s food choices and food intake, offering an innovative perspective of analysis that addresses the reality of children’s food packaging. Nevertheless, the results above show the need to increase the number of experimental studies in the field. Especially in that combined way, allowing comparative results about how the various elements behave in the presence of others. Even more, if these elements generate cognitive dissonance between them. The scarcity of experimental studies on issues of great social relevance is striking.

Furthermore, is necessary to improve the number and methodological quality of articles that study elements such as Colour or the use of Celebrities, given their social relevance. In the case of Colour, because of its importance for the food industry when designing the packaging of its products, as shown in content analysis and other research (50–54). And in the case of Celebrities, due to their significant increase in recent years, becoming a relevant phenomenon worldwide (55).

Finally, developing more experiments with food intake outcomes is needed in this area of research.

The number of articles in this study have been published in recent years with an increasing trend, which leads us to intuit the relevance of the subject. This is not surprising since it has been demonstrated that the different packaging elements are one of the obesogenic factors contributing to the increase in childhood obesity worldwide (4, 56). It is necessary to study them to continue understanding how they influence children’s food choices and food intake and, in general, their consumption and eating habits, trying to mitigate their effects with short-, medium- and long-term actions by governments and responsible organisations.

## Conclusion

Previous SR such as Smith et al. (15), Hallez et.al (17), and Elliot et al. (8) have addressed more or less directly the influence of packaging on children’s food choices and food intake. The work presented here extends and complements those previous studies. Based on experimental studies and considering their methodological quality, this SR provides a global perspective of the effect of Marketing Techniques on Packaging and Nutrition Labelling on children’s food choices and food intake.

The results show differences in the influence of different packaging elements on children’s food choices. It has been shown that most of the Marketing Techniques on Packaging and Nutrition Labelling affect children, where the Cartoon takes precedence over the other elements. However, most studies deal with the analysis of packaging elements in isolation. Few comparative studies allow us to conclude these elements’ influence when combined with others. As discussed above, more studies in this direction are needed. The results obtained can be of high scientific rigour thanks to the risk analysis of the articles used in this study.

Of particular interest is the lack of consistency in the studies found on the effect of Nutrition Labelling on children’s choice and intake, beyond the improvement in children’s knowledge of the product’s level of safety. Without new evidence, it is difficult to conclude the effectiveness of tools promoted and developed for some years by public authorities.

With its limitations, this work systematises the contrasting evidence on the power of influence of one of the marketing variables that has been pointed out as one of the causal factors of unhealthy eating habits in children. The results may be helpful for policymakers, programme managers, and health professionals when designing public policies aimed at improving children’s health and quality of life, seeking to prevent problems such as overweight and obesity, which are a real scourge in our times.

## Author contributions

AA-C, RA-P, and MM-S contributed to the conceptualisation and methodology of this research. AA-C and CO-S had searched for studies on databases and collaborated for peer review. AA-C had drafted the manuscript and prepared the figures and tables with the RA-P, MM-S, and CO-S assistance and supervision. All authors have contributed to the development of the article, writing, editing, read, and agreed to the published version of the manuscript.
